# Structure–Activity Relationship Study of Acyclic Terpenes in Blood Glucose Levels: Potential α-Glucosidase and Sodium Glucose Cotransporter (SGLT-1) Inhibitors

**DOI:** 10.3390/molecules24224020

**Published:** 2019-11-06

**Authors:** Miguel Valdes, Fernando Calzada, Jessica Mendieta-Wejebe

**Affiliations:** 1 Instituto Politécnico Nacional, Sección de Estudios de Posgrado e Investigación, Escuela Superior de Medicina, Plan de San Luis y Salvador Díaz Mirón S/N, Col. Casco de Santo Tomás, Ciudad de México CP 11340, CDMX, Mexico; 2 UMAE Hospital de Especialidades 2º Piso CORSE Centro Médico Nacional Siglo XXI, Instituto Mexicano del Seguro Social, Av. Cuauhtemoc 330, Col. Doctores, Ciudad de México CP 06720, CDMX, Mexico

**Keywords:** acyclic terpenes, antihyperglycemic activity, diabetes mellitus

## Abstract

Twelve terpenoids were evaluated in the treatment of type 2 diabetes mellitus: seven monoterpenes (geranyl acetate (**1**), geranic acid (**2**), citral (**3)**, geraniol (**4**), methyl geranate (**5**), nerol (**6**), and citronellic acid (**7**)), three sesquiterpenes (farnesal (**8**), farnesol (**9**), and farnesyl acetate (**10**)), one diterpene (geranylgeraniol (**11**)), and one triterpene (squalene (**12**)) were selected to carry out a study on normoglycemic and streptozotocin-induced diabetic mice. Among these, **2**, **3**, **7**, **8**, **9**, and **10** showed antihyperglycemic activity in streptozotocin-induced diabetic mice. They were then selected for evaluation in oral sucrose and lactose tolerance tests (OSTT and OLTT) as well as in an oral glucose tolerance test (OGTT). In the OSTT and OLTT, compounds **3**, **7**, **8**, **9**, and **10** showed a reduction in postprandial glucose peaks 2 h after a sucrose or lactose load (comparable to acarbose). In the case of the OGTT, **2**, **7**, **8**, **9**, and **10** showed a reduction in postprandial glucose peaks 2 h after a glucose load (comparable to canagliflozin). Our results suggest that the control of postprandial hyperglycemia may be mediated by the inhibition of disaccharide digestion, such as sucrose and lactose, and the regulation of the absorption of glucose. The first case could be associated with an ∝-glucosidase inhibitory effect and the second with an inhibition of the sodium–glucose type 1 (SGLT-1) cotransporter. Finally, five acyclic terpenes may be candidates for the development and search for new α-glucosidase and SGLT-1 cotransporter inhibitors.

## 1. Introduction

Diabetes mellitus (DM) is considered to be a group of metabolic diseases characterized by high glucose levels resulting from alterations in the metabolism of carbohydrates, lipids, and proteins. These alterations are generated by defects in insulin secretion, action, or both [1]. If patients do not control their glycemic levels, this can cause long-term damage or dysfunction in several organs, including the retinas, kidneys, nervous system, heart, and blood vessels, which in time becomes a deadly situation for the patient [2,3]. The World Health Organization (WHO) estimates that worldwide, from 1995 to the current day, the number of people living with DM has tripled, with an estimated value of more than 425 million people (8.8%), with a range age between 20 and 79 years [4,5]. There are several treatments for this disease, including sulfonylureas, meglitinides, biguanides, thiazolidinediones, glucagon-like peptide-1 (GLP-1) analogs, dipeptidyl peptidase-4 (DPP-4) inhibitors, and sodium–glucose cotransporter (SGLT) inhibitors [6,7,8,9].

One of the principal approaches for reducing postprandial hyperglycemia in patients with DM is the prevention of hydrolysis and the absorption of carbohydrates after food uptake [10]. Complex oligosaccharides and disaccharides, such as lactose and sucrose, must be broken down into individual monosaccharides before being absorbed in the duodenum and upper jejunum. This digestion is facilitated by enteric enzymes, including pancreatic α-amylase and α-glucosidase, which are attached to the brush border of intestinal cells [10]. The α-glucosidase inhibitors are useful drugs for reducing the high amount of glucose that patients with DM present with after food intake by inhibiting the hydrolysis of complex carbohydrates [11]. Acarbose and miglitol are the most commonly used ∝-glucosidase inhibitors. In the case of acarbose, molecular docking experiments have shown that it binds to certain amino acid residues (Phe 231, His 239, Asn 241, Ala 278, His 279, Gly 280, Thr 301, Glu 304, Pro 309, and Phe 310) that are located in the catalytic site of the enzyme, and this inhibits its catalytic activity in complex disaccharides and oligosaccharides [12]. Monosaccharides, such as glucose and fructose, can be transported out of the intestinal lumen into the bloodstream by SGLT1 and other glucose transport facilitating systems (GLUTs) [13]. Sodium glucose cotransporters (SGLTs) are transporters that couple the entrance of Na^+^ and glucose using the electrochemical gradient in favor of the entrance of Na^+^, transporting the glucose against a concentration gradient. The structure of SGLTs contains 14 transmembrane alpha-helix-type crosses with amino and carboxyl terminal groups on the extracellular side and a glycosylation site between segments 6 and 7. The transport of Na^+^ is carried out in a region near the amino terminal, and glucose enters a region near the carboxyl terminal. The Na^+^ transported inside the cells is pumped by Na^+^/K^+^ adenosine triphosphate-hydrolyzing enzimes (ATPase) through the basolateral membrane. The accumulated glucose in the epithelial cells is mobilized out of the cell by glucose transport facilitating systems (GLUTs) [13]. Sodium-glucose co-transporter 1 is highly expressed on the brush-border membrane of villus enterocytes in the proximal part of the small intestine, and it is responsible for dietary glucose absorption. Canagliflozin is a moderate SGLT1 inhibitor drug [14] generating a decrease in hyperglycemia levels in patients with DM, and it has been reported that the inhibition of SGLT1 in the small intestine favors a decrease in intestinal glucose absorption [15]. 

**Figure 1:**
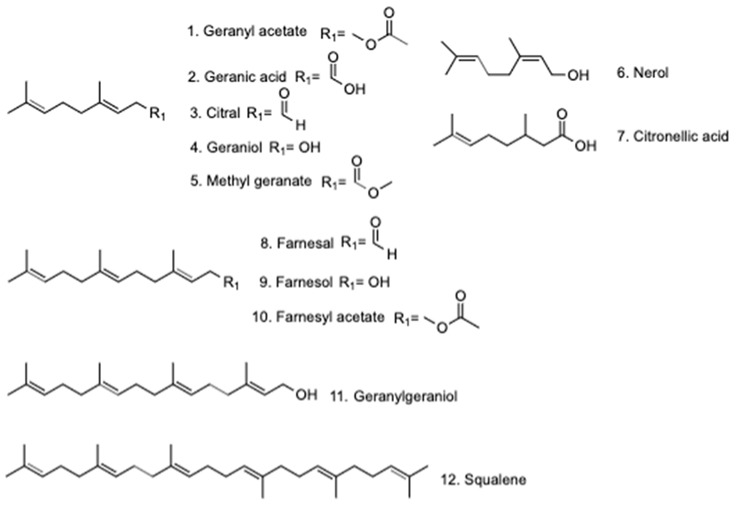
Structure of terpenes tested for activity in blood glucose levels: oral sucrose tolerance test (OSTT), oral lactose tolerance test (OLTT), and oral glucose tolerance test (OGTT) assays.

Terpenes such as monoterpenes and sesquiterpenes are compounds commonly found in essential oils [16]. The interest in the study of terpenes has been increasing because they have been shown to have multiple biological attributes that can be useful for the treatment of several diseases, including antifungal, antibacterial, antiviral, antitumor, antiparasitic, hypoglycemic, anti-inflammatory, and analgesic properties [16]. Farnesol (molecular formula C_15_ H_26_O; International Union of Pure and Applied Chemistry (IUPAC): 3,7,11-trimethyl-2,6,10-dodecatrien-1-ol), an alcohol found in essential oils, is a natural terpene formed by 15 carbos made in plant cells by farnesyl pyrophosphate dephosphorylation [16]. Farnesol has been shown to reduce serum triglyceride levels and prevent hyperglycemia and hepatic steatosis in obese animals with a high-fat diet [17]. Another important study on farnesol was carried out by Calzada et al., who showed that farnesol was a compound that can be isolated from the leaves of *Annona diversifolia* Safford, and it was shown that farnesol has antihyperglycemic activity in acute and subchronical assays, with the inhibition of the enzyme α-glucosidase being part of its action mechanism [18]. The significant in vivo antihyperglycemic activity displayed by farnesol prompted us to undertake the present investigation. The test compounds differed in terms of the number of carbons, the nature of substituent in C1, and the presence or absence of a 2,3-double bond (Figure 1): they were seven monoterpenes (geranyl acetate (**1**), geranic acid (**2**), citral (**3**), geraniol (**4**), methyl geranate (**5**), nerol (**6**), and citronellic acid (**7**)); three sesquiterpenes (farnesal (**8**), farnesol (**9**), and farnesyl acetate (**10**)); one diterpene (geranylgeraniol (**11**)); and one triterpene (squalene (**12**)). These compounds were selected to evaluate their activity in blood glucose levels in normoglycemic and streptozocin-induced diabetes type 2 mice (SID2). The compounds with significative activity in SID2 mice were evaluated in oral sucrose and lactose tolerance tests (OSTT and OLTT) and in an oral glucose tolerance test (OGTT). These tests may indicate that the control of postprandial glucose levels shown by the terpenoids tested might be an antihyperglycemic effect mediated by the regulation of glucose uptake from the intestinal lumen through the inhibition of complex carbohydrate digestion (OSTT and OLTT) or simple carbohydrate absorption (OGTT). In this sense, the retardment of the postprandial peak after a complex carbohydrate load (sucrose and lactose) or a simple carbohydrate (glucose) can be associated with the inhibition of intestinal α-glucosidase (OSTT and OLTT) or the inhibition of sodium–glucose cotransporter type 1 SGLT1 (OGTT) [19]. 

## 2. Results

### 2.1. Acute Evaluation of the Compounds in Normoglycemic Mice (NM) and SID2 Mice

An acute evaluation was carried out to observe the activity of the glycemia values of the compounds that we proposed for this work. The acute activity in normoglycemic and streptozocin-induced diabetes type 2 (SID2) mice after the administration of the compounds and pharmacological controls (acarbose, canagliflozin, glibenclamide, and pioglitazone) is shown in Table 1.

In an acute test using normoglycemic mice (NM), compound **4**, glibenclamide, and pioglitazone showed a decrease in glycemia values at 2 and 4 h; in the case of **5**, **6**, and **11**, there was activity at 4 h. The remaining compounds did not generate alterations in the glycemia values of NM treated with acarbose as well (Table 1).

In an acute assay using SID2 mice, **2**, **9**, and acarbose showed a significant decrease in hyperglycemic values at 2 h; **6**, **7**, **10**, and **11** showed a significant decrease in hyperglycemic values at 4 h. In the case of **3**, **5**, **8**, canagliflozin, glibenclamide, and pioglitazone, a significant decrease in hyperglycemic values was shown at 2 and 4 h, showing greater activity than acarbose, which worked only after 2 h of treatment (Table 1).

To continue the study, the compounds with a significant decrease in hyperglycemia values in SID2 mice (**2**, **3**, **7**, **8**, **9**, and **10**) were selected to be evaluated in an OSTT, OLTT, and OGTT. The compounds **5**, **6**, and **11**, as well as glibenclamide and pioglitazone, were discarded when continuing the study because they generated hypoglycemia in the NM, one of the main adverse effects that we were intending to avoid. In the case of glibenclamide and pioglitazone, they were discarded because their action mechanism is different than what we were evaluating, and the compounds **1**, **4**, and **12** were discarded because they did not show activity in terms of glycemic values.

### 2.2. Oral Sucrose and Lactose Tolerance Tests on Fasted Normoglycemic Mice (FNM)

In order to evaluate if the terpenes with activity in SID2 mice inhibited the postprandial hyperglycemic peaks after a complex carbohydrate load (as a possible α-glucosidase inhibitor), OSTT and OLTT assays were carried out. These tests were carried out using acarbose as a standard medication for this action mechanism. In the OSTT, all of the compounds as well as acarbose showed a reduction in the 2 h glycemic postprandial peak and significant activity with respect to sucrose control at 2 h and 4 h (Table 2).

In the OLTT, **2** presented a postprandial hyperglycemic peak at 2 h, and the remaining compounds as well as acarbose showed significant activity in inhibiting the postprandial peak at 2 h, which was significant in comparison to the lactose control (Table 2).

### 2.3. Oral Glucose Tolerance Test on Fasted Normoglycemic Mice (FNM)

The OGTT assay was carried out in order to determine if the terpenes evaluated reduced the absorption of glucose. This could be related to a selective inhibitory activity of sodium–glucose cotransporter type 1 (SGLT-1). After evaluation, compound **3** showed a significant increase in terms of blood glucose levels, and the remaining compounds as well as canagliflozin showed an inhibition of the postprandial peak of glycemia with significant values. In the case of canagliflozin, there was a significant decrease in the glycemia values below the normoglycemic values (Table 2).

## 3. Discussion

This work was carried out to evaluate the activity of 12 different terpenes in terms of glycemia values with the aim of searching for molecules with activity for DM treatment. Initially, the compounds **2**, **3**, **7**, **8**, **9**, and **10** at a dose of 50 mg/kg showed activity in the SID2 mice. Additionally, we observed that in terpenes with a higher quantity of carbon as well as squalene (**12**), the activity in terms of glycemic values was lost. This effect was also observed in geranyl acetate (**1)**, where we observed that monoterpenes with a presence of acetate groups in C1 completely lost their activity in terms of the blood glucose values. On the other hand, we observed that in monoterpenes and diterpenes, the hydroxyl group in C1, including geraniol (**4**), nerol (**6**), and geranyl geraniol (**11**), might have been an important requirement for the hypoglycemic activity observed in NM and SID2 mice. This hypoglycemic activity was also observed if the hydroxyl group was substituted by an acetate in C1 such as methyl geranate (**5**): we suggest that it is possible that these compounds act through an insulin-dependent mechanism such as sulfonylurea. These compounds were discarded when continuing the study because they generate hypoglycemia in NM, a side effect that we wanted to avoid. With respect to the products **2**, **3**, **7**, **8**, **9**, and **10**, they showed activity in terms of the hyperglycemic values. This activity was considered antihyperglycemic activity because when they were evaluated in NM mice, they did not generate hypoglycemia, and in SID2 they reduced the hyperglycemic levels [7,8,18]: this activity was similar to that observed with acarbose. The compounds **2**, **3**, **7**, **8**, **9**, and **10** were selected to continue the study. In the OSTT and OLTT, we observed that all of the compounds showed activity in avoiding the postprandial glycemic peak at 2 h. In the OLTT, compound **2** was the only compound that did not have activity in the postprandial peak. 

With these results, in the cases of **3**, **7**, **8**, **9**, and **10**, we suggest that the reduction of the postprandial peaks of glucose is mediated by the inhibition of the enzyme α-glucosidase in the small intestine. In the case of the monoterpene **2**, we propose that the presence of a carboxylic acid in C1, as indicated in Figure 1, helps the molecule avoid the hydrolysis of disaccharides with glycosidic bonds, such as type α-1,4 in sucrose [19]. In addition, we observed that the structure of **2** showed that this kind of compound was not active in enzymes dedicated to hydrolyzing the glycosidic bond type β-1,4 present in lactose [19].

When the oral glucose tolerance test (OGTT) was performed, we observed that compound **3** was not active in the hyperglycemic peak after a glucose load. In the cases of **2**, **7**, **8**, **9**, and **10**, they showed an inhibition of the postprandial glycemic peak, as did canagliflozin. It should be noted that canagliflozin is not a selective inhibitor of SGLT-1 cotransporters [14]: this drug acts mostly on SLGT-2 located at a renal level in the proximal contoured tubule [13,20]. SGLT-1 mediates almost all sodium-dependent glucose absorption in the small intestine, while in the kidney, SGLT-2 and (in smaller amounts) SGLT-1 represent more than 90% and almost 3%, respectively, of the absorption of glomerular ultrafiltrate glucose [15,21]. It is possible that this generates hypoglycemia in mice after the administration of canagliflozin. Table 1 is a result of the inhibition of both SGLT cotransporters. In the cases of **2**, **7**, **8**, **9**, and **10**, it is suggested that these compounds act selectively on SGLT-1, because when they were administered in NM, they did not generate hypoglycemia. In conclusion, the development of new molecules aimed at the selective inhibition of SGLT-1 is an excellent alternative to control the glycemia of patients with DM. In this sense, 5 molecules (**2**, **7**, **8**, **9**, and **10**) of the 12 used in this work are good candidates for the development and search for a new targeted treatment for the selective inhibition of SGLT-1 and joint activity as an α-glucosidase inhibitor. This investigation is the first step in the development of new molecules starting with the terpenes proposed; however, it is necessary to carry out more experiments to confirm the selective SGLT1 inhibition and enzyme α-glucosidase inhibition activity of these compounds. Once the proposed activities have been demonstrated, we can establish whether compounds alone or in combination with a standard medication show greater activity in order to develop new treatments for DM. 

## 4. Materials and Methods 

### 4.1. Chemicals

Geranyl acetate (≥97%, product number (PN): 173495-25G), citronellic acid (98%, PN: 303429-25ML), geranic acid (85%, PN: 427764-25ML), citral (95%, PN: C83007-100 ML), geraniol (98%, PN: 163333-25G), nerol (97%, PN: 268909-5ML), methyl geranate (PN: CDS001198), farnesol (95%, mixture of isomers, PN: F203-25G), farnesal (≥85%, mixture of isomers, PN: 46188-1ML-F), farnesyl acetate (technical, mixture of isomers, PN: 45895-10ML-F), geranylgeraniol (≥85%, PN: G3278-100MG), squalene (≥98%, PN: S3626-10ML), streptozocin (≥75% α-anomer basis, PN: S0130-5G), nicotinamide (≥99.5%, PN: 47865-U), glucose (anhydrous, PN: D9434-1Kg), sucrose (≥99.5%, PN: S9378-1Kg), pioglitazone (≥98%, PN: E6910-10MG), acarbose (PN: PHR1253-500MG), canagliflozin (95%, PN: 721174-1G), and glibenclamide (PN: PHR1287-1G) were purchased from Sigma-Aldrich^®^, (St. Louis, MO, United States). Buffer solution (citric acid/sodium hydroxide/hydrogen chloride, pH 4.00, PN: 109445) was purchased from Merck^®^ (Darmstadt, Germany), and saline solution 0.9% (solution 1000 mL) was purchased from PISA^®^ pharmaceutics (Mexico City, Mexico).

For the antihyperglycemic activity, oral glucose, sucrose, and lactose tolerance tests, Balb/c male mice aged 8–10 weeks (25 ± 5 g) with glucose level values of 150 ± 10 mg/dL were used. All of the animals were raised in the Animal House of the National Medical Center “Siglo XXI” at Instituto Mexicano del Seguro social (IMSS). Investigations using experimental animals were conducted in accordance with the Official Mexican NOM0062-ZOO-1999 [22] for Animal Experimentation and Care. The mice were maintained at room temperature (22 ± 2 °C) on a 12-h light–dark natural cycle. Mice were fed with a standard diet and water ad libitum. All assays were conducted with the approval of the Specialty Hospital Ethical Committee of the National Medical Center “Siglo XXI” at IMSS (register: R-2015-3601-211 and R-2019-3601-004). 

### 4.2. Induction of Experimental Type 2 Diabetes in Mice

Streptozocin-induced type 2 diabetes (SID2) was induced in BALB/c mice with a streptozocin/nicotinamide model (STZ/NA). In accordance with the procedure described in Jeng-Dong et al. [15], mice fasted for 16 h before receiving the treatment (day 0). Streptozocin (STZ) was dissolved in a cold pH 4 buffer solution, and this was administered at 100 mg/kg by intraperitoneal injection twice on days 1 and 3. Nicotinamide (NA) was dissolved in a cold saline solution and administered at 240 mg/kg by intraperitoneal injection 30 min after the administration of STZ on day 1 [23]. At the end of day 3, a high-carbohydrate diet solution was used (sucrose 10% solution) ad libitum over three days to induce experimental type 2 diabetes in STZ/NA mice. On day 5, the high-carb solution was retired and substituted with water at libitum: 24 h later, the development of SID2 was determined by measuring postprandial blood glucose levels using the glucose oxidase method (ACCU-CHECK^®^ Performa Blood Glucose System, Roche^®^, DC, Mexico). Additionally, β-cell function was evaluated through the administration of glibenclamide and by measuring the decrease of glucose values after administration [18,23].

### 4.3. Acute Antihyperglycemic Evaluation of the Terpenes

Animals were randomly divided into 26 groups (*n* = 6 animals per group) as follows: normoglycemic mice (NM) and SID2 mice, both treated with vehicle (2% tween 80 in water) (17 groups of NM and 17 of SID2 mice treated with geranyl acetate (**1**), geranic acid (**2**), citral (**3**), geraniol (**4**), methyl geranate (**5**), nerol (**6**), citronellic acid (**7**), farnesal (**8**), farnesol (**9**) farnesyl acetate (**10**), geranylgeraniol (**11**), and squalene (**12**) (50 mg/kg)). In order to compare the pharmacological effect of the standard medication, acarbose, canagliflozin, glibenclamide, and pioglitazone were administered at 50 mg/kg. All of the compounds were solubilized in 2% tween 80 in water and were administered orally in a 0.5 mL volume per mouse. Blood samples were collected from the tail vein before (0 h), 2 h, and 4 h after administration, and samples were assessed using the glucose oxidase method, as previously mentioned [18]. 

### 4.4. Oral Sucrose and Lactose Tolerance Tests of Terpenes and Aacarbose in Fasted Normoglycemic Mice (FNM)

The potential α-lucosidase inhibitory activity of the compounds was measured using oral sucrose and lactose tolerance tests (OSTT and OLTT). This was performed in NM fasted male (FNM) BALB/c mice under 20–25 g, which were randomly divided into 12 groups (*n* = 6 animals per group) as follows: a control group (FNM Control) treated with vehicle (2% tween 80 in water); a group treated with sucrose or lactose at 3 g/kg (FNM + S, FNM + L); 9 groups treated with geranic acid (**2**), citral (**3**), citronellic acid (**7**), farnesal (**8**), farnesol (**9**), and farnesyl acetate (**10**) (50 mg/kg); and a group treated with acarbose (50 mg/kg), an α-glucosidase inhibitor, which was used as a pharmacological control. All of the treatments were solubilized as previously mentioned 30 min after the administration of the treatments, and a sucrose or lactose load (3 g/kg) was administered to the mice. Glycemia values were determined at 2 and 4 h after the administration of the carbohydrate using the glucose oxidase method [12].

### 4.5. Oral Glucose Tolerance Test of Terpenes and Canagliflozin in Fasted Normoglycemic Mice (FNM)

The possible selective sodium–glucose cotransporter 1 (SGLT-1) activity of the compounds was measured using oral glucose and lactose tolerance tests (OGTT). This assay was carried out under the same conditions as the OSTT and OLTT, but in this case, a glucose load (1.5 g/kg) was given to fasted NM, and canagliflozin (50 mg/kg), a moderate SGLT-1 inhibitor [14], was used as a pharmacological control. Glycemia values were determined at 2 and 4 h after the administration of the carbohydrate for the OSTT and OLTT using the glucose oxidase method [18].

### 4.6. Statistical Analysis

All of the results are expressed as the mean values ± standard error of the mean. All statistical analyses were performed by using GraphPad Prism version 6.0 for Macintosh (GraphPad software, San Diego, CA, USA). The statistical evaluation was carried out through an analysis of variance followed by a Bonferroni test for multiple comparisons. *p* < 0.05 was considered a statistically significant difference between the group means.

## 5. Conclusions

In short, in this study on the relationship between structure and activity, 12 terpenes were proposed, and it was demonstrated which of them could be good candidates for activity in terms of hyperglycemia values. Additionally, we suggest that this activity in terms of hyperglycemia values is partially mediated by inhibition of the α-glucosidase enzyme and that some of the terpenes may have selectivity for the SGLT-1 cotransporter, reducing glucose absorption. However, more experiments are needed to confirm this activity. 

## Figures and Tables

**Table 1:** Effect of a single oral administration of the compounds on the blood glucose levels of normoglycemic mice (NM) and streptozocin-induced type 2 diabetic mice (SID2).

Treatment	Glycemia (mg/dL)		
	0 h	2 h	4 h
NM Control	142.6 ± 7.3	132 ± 5.5	135 ± 5.8
NM + Geranyl Acetate (**1**)	150 ± 3.5	171 ± 13.6	145.3 ± 14.6
NM + Geranic Acid (**2**)	143.3 ± 6.1	157.3 ± 8.4	134 ± 0.5
NM + Citral (**3**)	145.6 ± 7.3	138 ± 4.1	136 ± 6.5
NM + Geraniol (**4**)	143 ± 5.1	117 ± 10.4^*^	110.3 ± 12.8^*^
NM + Methyl Geranate (**5**)	149.3 ± 2	167 ± 2.2	131 ± 1.7^*^
NM + Nerol (**6**)	143.3 ± 5.8	140 ± 6.2	124 ± 8.7^*^
NM + Citronellic Acid (**7**)	142 ± 6.6	139 ± 7	138 ± 11
NM + Farnesal (**8**)	148.3 ± 3.4	157.3 ± 13.7	147.3 ± 1.2
NM + Farnesol (**9**)	136 ± 6.6	130.6 ± 5.2	130 ± 5.5
NM + Farnesyl Acetate (**10**)	150 ± 3.6	150.6 ± 14.8	153.6 ± 12.1
NM + Geranylgeraniol (**11**)	147.3 ± 5	153.3 ± 12.7	116.5 ± 10.1^*^
NM + Squalene (**12**)	154.3 ± 2	150.6 ± 19.7	148 ± 19
NM + Acarbose	144 ± 4	145 ± 2	170 ± 19
NM + Canagliflozin	131.8 ± 6.9	101.6 ± 5.1^*,•^	101 ± 9.3^*,••^
NM + Glibenclamide	148.6 ± 0.3	98.6 ± 5.7^*,•^	119 ± 13.6^*,••^
NM + Pioglitazone	150.3 ± 3.8	116.5 ± 1.5^*,•^	125.6 ± 1.9^*,••^
SID2 Control	330.3 ± 20.7	368.7 ± 20.2	352.2 ± 15.1
SID2 + Geranyl Acetate (**1**)	356.3 ± 20.5	337.3 ± 23.1	358 ± 23
SID2 + Geranic Acid (**2**)	362.3 ± 15.3	220.5 ± 3.4^*, ¥^	346.3 ± 41
SID2 + Citral (**3**)	369 ± 13	263.6 ± 32^*, ¥^	248 ± 22^*, ¥¥^
SID2 + Geraniol (**4**)	364 ± 10	419.3 ± 33	302 ± 45.1
SID2 + Methyl Geranate (**5**)	334.6 ± 5.1	238.4 ± 17^*, ¥^	244.6 ± 18^*, ¥¥^
SID2 + Nerol (**6**)	368.3 ± 4.5	333.1 ± 25	215.7 ± 17^*, ¥¥^
SID2 + Citronellic Acid (**7**)	378.6 ± 10	329.3 ± 10.3^*^	365.6 ± 12.8
SID2 + Farnesal (**8**)	336.5 ± 9.3	260.3 ± 10^*, ¥^	243.3 ± 20.6^*, ¥¥^
SID2 + Farnesol (**9**)	339.6 ± 10.3	305.3 ± 7.7^*, ¥^	337 ± 7.2
SID2 + Farnesyl Acetate (**10**)	357.6 ± 27	306 ± 20.2	288 ± 16.5^*, ¥¥^
SID2 + Geranylgeraniol (**11**)	361.3 ± 16	303 ± 16.2	292.5 ± 22.3^*^
SID2 + Squalene (**12**)	352 ± 24	321.6 ± 17.6	323.3 ± 33.8
SID2 + Acarbose	337.7 ± 22.9	196.8 ± 12.6^*,¥^	335.5 ± 25
SID2 + Canagliflozin	367.3 ± 5.94	157.6 ± 22.2^*, ¥^	102 ± 8.1^*, ¥¥^
SID2 + Glibenclamide	357 ± 7.5	271 ± 6^*, ¥^	201 ± 10.9^*, ¥¥^
SID2 + Pioglitazone	350.3 ± 5.4	245 ± 28.2^*, ¥^	240 ± 13.8^*, ¥¥^

All treatments were administered at 50 mg/kg. Data are expressed as means ± SEM, *n* = 6; * *p* < 0.05 versus initial values; ^•^ *p* < 0.05 vs. NM control for 2 h; ^••^ *p* < 0.05 vs. NM control for 4 h; ^¥^ *p* < 0.05 vs. SID2 control for 2 h; ^¥¥^ *p* < 0.05 vs. SID2 control for 4 h. SEM: standard error of the mean; NM: normoglycemic mice; SID2: streptozocin-induced diabetes 2 mice. Acarbose, canalgiflozin, glibenclamide, and pioglitazone were used as pharmacological controls.

**Table 2:** Effect of terpenes on oral sucrose, lactose, and glucose tolerance tests.

Treatment	Glycemia (mg/dL)		
	0 h	2 h	4 h
FNM Control	106.3 ± 4	103 ± 4	107.3 ± 3.7
FNM + S (3g/kg)	104.3 ± 2	158.6 ± 7.4^*, ∆^	135.3 ± 7^*, ∆∆^
FNM + S + Geranic Acid (**2**)	112 ± 2.6	108 ± 2.5^†^	115.6 ± 10^††^
FNM + S + Citral (**3**)	109.6 ± 3.5	127 ± 4.3^*, †^	100 ± 5.8^††^
FNM + S + Citronellic Acid (**7**)	108.3 ± 3.4	93 ± 8.3^†^	92 ± 10.4^††^
FNM + S + Farnesal (**8**)	112.3 ± 2.9	121.3 ± 9^†^	108.6 ± 10.9^††^
FNM + S + Farnesol (**9**)	114.6 ± 3.4	104.2 ± 5.6^†^	108 ± 1.7^††^
FNM + S + Farnesyl Acetate (**10**)	109 ± 5.5	110.6 ± 11.9^†^	99.3 ± 4.6^††^
FNM + S + Acarbose (50 mg/kg)	110 ± 2.3	113 ± 7.5^†^	113.3 ± 8.5^††^
FNM + L (3g/kg)	104.3 ± 0.3	156 ± 14^*, ∆^	99.3 ± 8.9
FNM + L + Geranic Acid (**2**)	114.6 ± 1.4	139 ± 5.4^*, ∆^	149.6 ± 8^*, ∆∆, ¥¥^
FNM + L + Citral (**3**)	114.3 ± 2.4	118.6 ± 7.6^¥^	120 ± 4
FNM + L + Citronellic Acid (**7**)	101-6 ± 5.2	101 ± 4^¥^	88.3 ± 13.7
FNM + L + Farnesal (**8**)	110.6 ± 5.2	125 ± 1^*, ∆, ¥^	110.3 ± 1.1
FNM + L + Farnesol (**9**)	111.6 ± 3.7	122.3 ± 9.6 ^¥^	105.6 ± 7.4
FNM + L + Farnesyl Acetate (**10**)	104.3 ± 7.3	118.3 ± 12.3^¥^	88.6 ± 5.6
FNM + L + Acarbose (50 mg/kg)	109.3 ± 3.7	108.3 ± 11.3^¥^	111.6 ± 2.9
FNM + G (1.5g/kg)	109.3 ± 8.1	155.6 ± 5.8^*, ∆^	109.6 ± 7.4
FNM + G + Geranic Acid (**2**) 2y4	118.3 ± 6.3	116.6 ± 14.2^•^	88.6 ± 9.9
FNM + G + Citral (**3**)	113.3 ± 5.3	158 ± 13.8^*, ∆^	106.6 ± 6.3
FNM + G + Citronellic Acid (**7**)	113 ± 3.4	111.3 ± 0.8^•^	104.3 ± 2.02
FNM + G + Farnesal (**8**)	112 ± 0.5	120.3 ± 1.45^•^	109.3 ± 6
FNM + G + Farnesol (**9**)	102.6 ± 6	113.3 ± 8.9^•^	96 ± 6.8
FNM + G + Farnesyl Acetate (**10**)	116.3 ± 1.2	129 ± 5.1^•^	96.3 ± 2.9
FNM + G + Canagliflozin (50 mg/kg)	107.3 ± 2.9	83.3 ± 7^*, ∆, •^	76.6 ± 3.2^*, ∆∆, ••^

All treatments were administered at 50 mg/kg, glucose was administered at 1.5 g/kg, and sucrose and lactose were administered at 3 g/kg. Data are expressed as means ± SEM, *n* = 6; * *p* < 0.05 vs. initial values; ^∆^ *p* < 0.05 vs. NM control for 2 h; ^∆∆^ *p* < 0.05 vs. NM control for 4 h; ^•^ *p* < 0.05 vs. FNM + G control for 2 h; ^••^ *p* < 0.05 vs. FNM + G control for 4 h; ^†^ *p* < 0.05 vs. FNM + S control for 2 h; ^††^ *p* < 0.05 vs. FNM + S control for 4 h; ^¥^ *p* < 0.05 vs. FNM + L control for 2 h; ^¥¥^ *p* < 0.05 vs. FNM + L control for 4 h. SEM: standard error of the mean; FNM: fasting normoglycemic mice; G: glucose; S: sucrose: L: lactose.
